# National Guidelines for Public Health Emergency Surveillance within the Brazilian Unified Health System

**DOI:** 10.1590/S2237-96222026v35e20260071.en

**Published:** 2026-10-12

**Authors:** Raquel Proença, Camila Cunha Maia Nogueira Nunes, Alessandro Pecego Martins Romano, Camila Pinto da Silva, Carlos Henrique Michiles Frank, Felipe Soares de Moraes, Francisco Silvanei dos Santos Gonçalves, Gabriel dos Santos Bandeira, Igor de Assis Rodrigues, João Roberto Cavalcante, Jose Lucas Pinho da Fonseca, Lucio Campello Silva, Magda Machado Saraiva Duarte, Maiara Almeida Maia, Mariana Sanches de Mello, Marina Pissurno do Nascimento, Otto Henrique Nienov, Paula Orofino Moura Costa, Rebeca Cristine Campos Martins, Sabrina Araújo de Melo, Vanessa de Paula Ferreira, Vinícius de Souza Casaroto, Daniel Roberto Coradi de Freitas, Taynná Vernalha Rocha Almeida, Marcio Henrique de Oliveira Garcia, Edenilo Baltazar Barreira

**Affiliations:** 1 Ministério da Saúde, Departamento de Emergências em Saúde Pública, Brasília, DF, Brazil

**Keywords:** Public Health Emergencies, Public Health, Public Health Surveillance, Unified Health System, Guidelines, Emergencias en Salud Pública, Salud Pública, Vigilancia en Salud Pública, Sistema Único de Salud, Guías

## Abstract

**Objective:**

To describe the national guidelines for public health emergency surveillance within the Brazilian Unified Health System (*Sistema Único de Saúde*, SUS).

**Guidelines:**

The increasing frequency of health, environmental, and technological events has led to more public health emergencies, requiring systems capable of early threat detection, risk assessment, and timely response. Public health emergency surveillance is an integral component of health surveillance and encompasses strategies such as indicator-based, event-based, hospital-based epidemiological, mass gathering, wastewater, community-based, and participatory surveillance. These strategies are complementary and should be supported by continuous monitoring and systematic risk assessment, while incorporating multisectoral approaches, risk communication, and One Health. Strengthening public health emergency surveillance involves integrating information systems, building workforce capacity, institutionalizing contingency plans and standard operating procedures, activating situation rooms and emergency operations centers, conducting timely outbreak investigations, communicating risks, and conducting intra-action and after-action reviews. Factors such as territorial inequalities, social vulnerabilities, and population-specific characteristics influence risk exposure, detection capacity, and the impacts of emergencies, underscoring the need to incorporate equity into surveillance explicitly.

**Conclusion:**

The guidelines help consolidate public health emergency surveillance as a core component of preparedness and response within the Unified Health System, reinforcing participatory, integrated, and equity-oriented approaches to protect health and reduce harm.

## Resumen

**Objetivo:**

Describir las directrices nacionales para la vigilancia de las emergencias en salud pública en el ámbito del Sistema Único de Salud (*Sistema Único de Saúde*, SUS).

**Directrices:**

La intensificación de los eventos sanitarios, ambientales y tecnológicos ha incrementado la ocurrencia de emergencias en salud pública, lo que exige sistemas capaces de detectar tempranamente las amenazas, evaluar los riesgos y movilizar respuestas oportunas. La vigilancia de las emergencias forma parte del campo de la vigilancia en salud e incluye estrategias como la vigilancia basada en indicadores, la vigilancia basada en eventos, la vigilancia epidemiológica hospitalaria, la vigilancia de eventos de concentración masiva, la vigilancia de aguas residuales, la vigilancia de eventos basada en la comunidad y la vigilancia participativa. Estas estrategias son complementarias y deben ir acompañadas de un monitoreo continuo y de una evaluación sistemática del riesgo, además de incorporar enfoques multisectoriales, de comunicación y de Una Sola Salud. El fortalecimiento de la vigilancia de las emergencias implica la integración de los sistemas de información, el fortalecimiento de las capacidades del personal de salud, la institucionalización de planes de contingencia y procedimientos operativos, la activación de salas de situación y centros de operaciones, la investigación oportuna de brotes, la comunicación de riesgos y la realización de evaluaciones durante y después del evento. Elementos como las desigualdades territoriales, las vulnerabilidades sociales y las especificidades poblacionales influyen en la exposición al riesgo, la capacidad de detección y los impactos de las emergencias, lo que exige la incorporación explícita de la equidad en la vigilancia.

**Conclusión:**

Las directrices contribuyen a consolidar la vigilancia de las emergencias como un componente estructurante de la preparación y la respuesta del Sistema Único de Salud, reforzando enfoques participativos, integrados y orientados a la equidad para proteger la salud y reducir los daños.

## Introduction 

Populations are continuously at risk of diseases and health conditions that may trigger outbreaks, epidemics, and pandemics, as well as potential disasters and disruptions in the provision of health services to the population. The national guidance for public health emergency surveillance aims to support decision-making to strengthen preparedness, early warning, and the incorporation of new strategies, while fulfilling the commitments established under the International Health Regulations^1^ ^,^ ^2^.

The frequency, scale, and complexity of emergencies have increased, driven by environmental impacts and climate change, contributing to the emergence and re-emergence of diseases, as well as to disasters and extreme events^3^. This context may be exacerbated by disruptions in health service delivery resulting from weakened or overburdened health systems, further aggravating health, economic, and social inequalities^3^. 

Public health emergency surveillance is an integral component of health surveillance and corresponds to the continuous and systematic process of detecting, verifying, monitoring, conducting risk assessment, and communicating risks related to public health events that may constitute potential emergencies^4^. This surveillance requires a multisectoral approach directed toward diseases, health conditions, and public health events that pose the greatest risk to the territory. It should consider local specificities, including the epidemiological profile, socioeconomic factors, population distribution and structure, and other elements relevant to the One Health approach. 

The importance of establishing specific surveillance strategies that target populations directly exposed to and affected by emergencies, as well as those in situations of vulnerability, should be emphasized^4^. In the context of emergencies, measures to prevent, control, and contain risks, harm, and adverse public health events should be established through a continuous process within the Brazilian Unified Health System (*Sistema Único de Saúde*, SUS) at all levels of management^4^.

The objectives of public health emergency surveillance are to detect, verify, and notify public health events early; assess risks and monitor events using an all-hazards approach; establish rapid communication flows in response to potential emergencies; and coordinate tripartite SUS cooperation strategies, promoting the implementation of the National Health Surveillance Policy (*Política Nacional de Vigilância em Saúde*, PNVS) in public health emergency surveillance activities^4^.

Gaps in surveillance at the local and global levels leave the world vulnerable. To address these vulnerabilities, collaborative health surveillance has emerged as a new approach that combines traditional techniques with innovative initiatives to protect health^3^. In Brazil, the collaborative model has been progressively incorporated through the integration of various strategies, including syndromic, genomic, and wastewater surveillance, as well as rumor monitoring. Early detection enables public health events to be rapidly controlled, thereby mitigating risks and preventing potential emergencies^2^. 

This article aimed to describe the national guidelines for public health emergency surveillance.

## Principles of the SUS and Conceptual Framework 

The guidelines presented herein were based on the principles and guidelines that inform the organization and operation of the SUS. Particular emphasis was placed on universality, comprehensiveness care, and equity, which guide the formulation of public policies and the organization of health actions, including those related to emergency preparedness, surveillance, and response^5^. 

Universality ensures access to health actions and services for the entire population; comprehensiveness care presupposes the integration of actions that broadly address health needs; and equity guides the prioritization of populations and territories in situations of vulnerability, recognizing the social, economic, and cultural inequalities that affect health^5^. These principles should be reflected transversally across public health emergency surveillance strategies, guiding activities from early detection through response and recovery.

The two main public health emergency surveillance strategies are indicator-based surveillance and event-based surveillance (Figure 1)^2^ ^,^ ^4^ ^,^ ^6^ ^,^ ^7^. Indicator-based surveillance is a public health monitoring strategy that uses quantitative indicators to assess population health, enabling early outbreak detection, epidemiological trend identification, and evaluation of the effectiveness of public health interventions^8^. 

Event-based surveillance is an early detection strategy that involves the rapid and continuous collection, assessment, and interpretation of unstructured information to detect public health events or situations that may pose a risk and that are generally not detected or are identified later by traditional information systems^2^ ^,^ ^6^ ^,^ ^7^. This information includes rumors originating from formal (official) sources, such as official reports from health services, and informal (unofficial) sources, such as news disseminated through the media, communities, and non-governmental organizations^2^ ^,^ ^6^ ^,^ ^7^. 

Mass gathering surveillance is conducted during large concentrations of people, such as popular festivals, sporting competitions, concerts, and other large-scale events. Monitoring these events involves a series of actions aimed at the surveillance and control of health risks and seeks to ensure a rapid and effective response in emergencies^9^ ^,^ ^10^.

**Figure 1:**
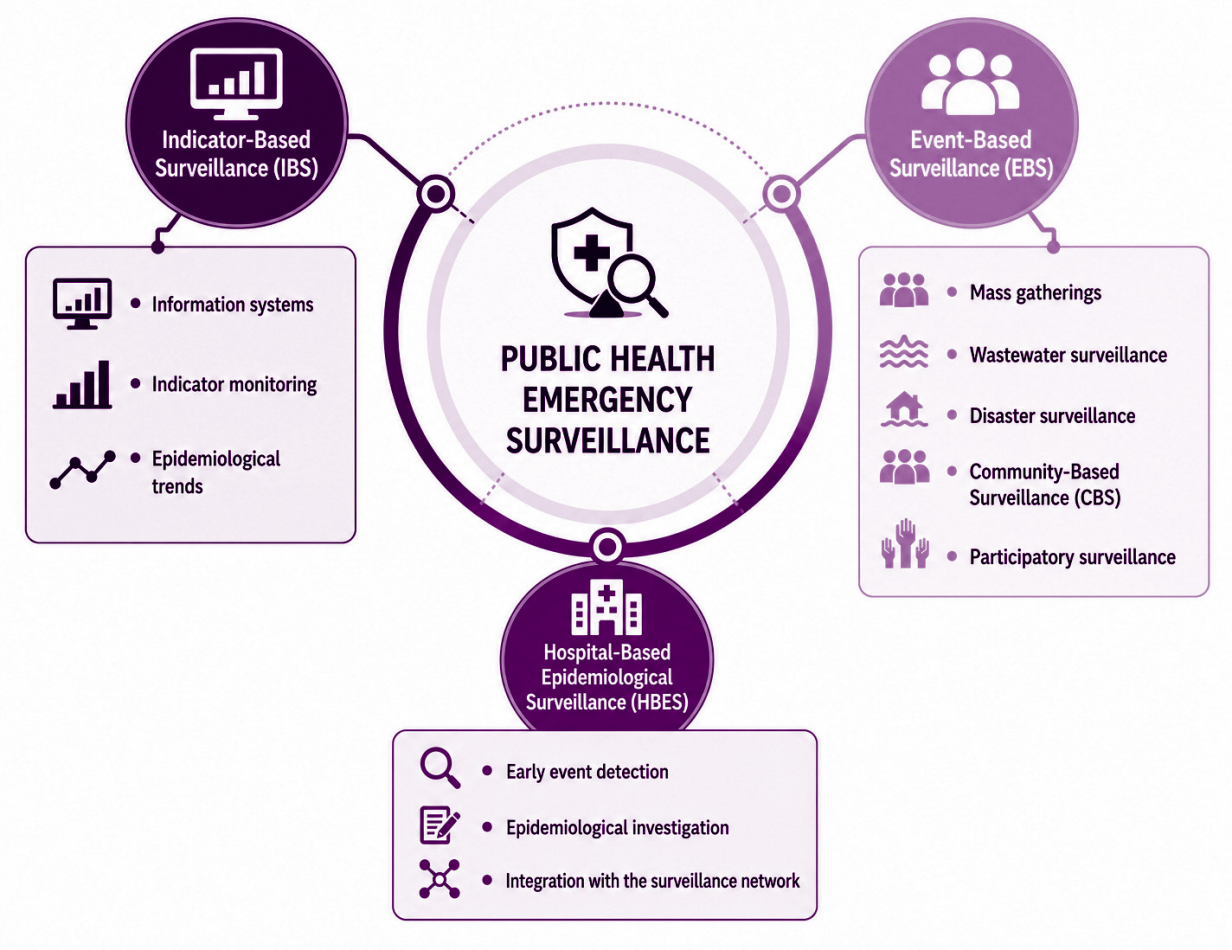
Components of public health emergency surveillance Brazil, 2026

Wastewater surveillance in the context of emergencies enables population-level monitoring of pathogens through wastewater analysis. Wastewater refers to freshwater that has been discharged after use in a variety of human activities, including household, commercial, industrial, and municipal services, as well as stormwater^11^. 

Community-based event surveillance consists of a participatory approach to health surveillance aimed at identifying and early communication of signs, rumors, or public health events, based on the actions of members of the community itself^12^. Grounded in the recognition of local knowledge and community perceptions of unusual changes in everyday life, this strategy enables the detection of information that is often not detected by formal surveillance systems.

Participatory surveillance refers to health monitoring in which the population plays an active role in providing information from their territories^13^. The population is the first to observe and experience the effects of diseases and health conditions, enabling the rapid reporting of early signals to health authorities and facilitating a more timely and proportionate response.

Hospital-based epidemiological surveillance emerged from the need to decentralize surveillance activities within healthcare facilities through Hospital Epidemiology Centers (*Núcleos Hospitalares de Epidemiologia*, NHE). Diseases, health conditions, and public health events identified in healthcare services should be promptly communicated by these units to the other networks, sectors, and services responsible for epidemiological surveillance activities at the local and national levels.

The main concepts related to public health emergencies are presented in Table 1.

**Table 1:** Key concepts of public health emergencies in the context of the national guidelines for public health emergency surveillance within the Brazilian Unified Health System (*Sistema Único de Saúde*, SUS)

Concept	Definition
Public health event^40^	A situation that may constitute a potential threat to public health, such as the occurrence of an outbreak or epidemic, a disease or health condition of unknown cause, or a change in the clinical or epidemiological pattern of known diseases. The assessment considers the potential for spread, magnitude, seriousness, severity, public health significance, and vulnerability, as well as epizootics and health conditions resulting from disasters or accidents.
Public health emergency^41^	A situation requiring the urgent implementation of measures for the prevention, control, and containment of risks, harm, and threats to public health.
Public Health Emergency of National Importance (PHENC)^41^	A public health emergency that, pursuant to Decree No. 7,616 of 17 November 2011, may be declared as a result of epidemiological events, disasters, or disruptions in the provision of health services to the population.
Public Health Emergency of International Concern (PHEIC)^1^	An extraordinary event that, under the International Health Regulations, is determined to: constitute a public health risk to other States through the international spread of disease; and potentially require a coordinated international response.
Outbreak^29^	A situation in which the number of cases of a disease, health condition, or public health event exceeds the expected level within a specific area or group of people during a given period.
Epidemic^29^	A situation in which a disease affects a large number of people and extends over a wide geographic area.
Pandemic^42^	An epidemic occurring worldwide or over a very wide area, crossing international borders and usually affecting a large number of people.
Natural disasters^43^	Events caused by natural processes or phenomena (hydrological, climatological, meteorological, or geological) that may result in loss of life or other health impacts, environmental and property damage, disruption of services, and social and economic disturbances.
Technological disasters^43^	Events resulting from technological or industrial conditions involving chemical, biological, radiological, or nuclear agents. They include accidents, incidents, or specific human activities that may result in loss of life or other health impacts, as well as environmental and property damage, disruption of services, and social and economic disturbances. They may occur intentionally or unintentionally.
Disruption in the provision of health care^41^	An event that, once formally recognized through the declaration of a state of emergency or public calamity by the affected federative entity, places the health of the population at risk because health services are unable to meet demand and exceeds the response capacity of the state and municipal health authorities within the Brazilian Unified Health System (Sistema Único de Saúde, SUS).

## Guidelines 

The guidelines presented below are based on the principles and guidelines of the SUS, which are expressed transversally across the different public health emergency surveillance strategies.

Indicator-based surveillance

Indicator-based surveillance is based on using indicators as summary measures of the population's health status and the performance of health services, ranging from the direct counting of cases to the calculation of proportions, ratios, and rates^14^. It can be applied to outbreak detection by monitoring indicators that support identification and rapid response; to the evaluation of health programs by measuring interventions; to health service planning by informing resource allocation and the distribution of health services based on identified needs; and to epidemiological surveillance by monitoring disease prevalence, incidence, and associated determinants. 

**Table 2:** Stages of the indicator-based surveillance cycle in the context of the national guidelines for public health emergency surveillance within the Brazilian Unified Health System (*Sistema Único de Saúde*, SUS)

Stage	Components
Data collection	Identification and collection of data (information systems, records, and other sources). Ensuring data quality, completeness, and timeliness.
Data analysis	Data processing. Identification of patterns, trends, and anomalies. Use of appropriate statistical methods and analytical software.
Interpretation	Interpretation of findings within the epidemiological context. Accounting for local factors and health policies. Critical analysis of the results.
Dissemination	Communication of findings to the surveillance network and health managers. Sharing of findings with health professionals and, when appropriate, the public. Clear and timely technical communication.

This surveillance follows a continuous cycle of data collection, analysis, interpretation, and dissemination (Table 2). Data may be collected from a variety of sources, including hospital records, health information systems, population-based surveys, and disease notification systems. Additionally, indicators related to the capacity of health services, such as hospital bed occupancy rates and demand for healthcare services, should be considered, as they may signal situations of risk of disruption in the provision of health services.

Event-based surveillance

Event-based surveillance is organized around a workflow that structures the management of detected signals until they are transformed into verified and actionable information. This workflow comprises five stages: detection, triage, assessment, verification, and dissemination (Table 3).

Event-based surveillance may be implemented through different modalities, all of which preserve the core stages described above while expanding surveillance coverage and strengthening the timely identification of public health events.

Natural and technological disasters

In the context of natural and technological disasters, event-based surveillance should monitor data, information, and risk alerts, such as weather forecasts. Records from civil protection and defense agencies and environmental institutions should be considered, in addition to monitoring disaster-prone areas, exposed populations, and the health service network within these territories. 

Monitoring the strategic stockpile of medicines, supplies, and equipment that may be mobilized during emergencies is part of this process, as are the establishment of rumor monitoring mechanisms and the dissemination of alerts to the competent authorities. Territorial characterization enables the identification of risks associated with flash floods, floods, mass movements, and failures of dams or critical infrastructure. Disaster health impact assessment, situation analysis of communicable and noncommunicable diseases, and the characterization of affected populations in situations of vulnerability are central components.

In chemical, biological, radiological, and nuclear emergencies, event-based surveillance includes detecting events that may trigger public health emergencies, characterizing territories, and identifying risk factors associated with these hazards. It also involves mapping referral hospitals and healthcare networks, as well as intersectoral coordination to ensure timely and integrated responses. It includes rumor monitoring, impact assessment, data analysis, characterization of affected populations, and assessment of the health situation.

**Table 3:** Stages of the event-based surveillance cycle in the context of the national guidelines for public health emergency surveillance within the Brazilian Unified Health System (*Sistema Único de Saúde*, SUS)

Stage	Description	Tool/process
Detection	Identification of "signals"^a^ through websites and internet search tools, media reports, and newspaper articles.	Epidemic Intelligence from Open Sources
Screening	Initial screening of the information collected for preliminary assessment.	Review of the headline and content of the report to identify: if there is information indicating changes in the clinical and epidemiological profile of a known disease; if there are reports of irregularities involving products and services; the possible emergence of an unusual or unknown disease; animal health events that may affect human health; and natural or technological disasters with the potential to affect public health or cause disruption in provision of health care.
Assessment	Assessment of the collected information and its classification according to relevance. Determination of whether a rumor has the potential to become a public health emergency.	Classification as relevant or not relevant, based on: geographical extent; characteristics of the reported rumor; healthcare service capacity; and public health significance and response capacity.
Verification	For rumors classified as relevant: verification of their validity; and collection of additional relevant information from official sources. Once a signal is verified, it becomes an "event." Events should trigger or prompt an appropriate level of response, including investigation and implementation of control measures.	Local rumors: verified with local stakeholders, such as technical units and institutions. Local rumors: verified with local stakeholders, such as technical units and institutions. National rumors: verified with the technical units responsible for the disease, health condition, or public health event, and with the units of Center for Strategic Information on Health Surveillance Network (*Rede Nacional dos Centros de Informações Estratégicas em Vigilância em Saúde*, CIEVS). International rumors: the National Cievs, acting as the operational center for the National Focal Point for the International Health Regulations, verifies the information or requests additional information from the World Health Organization member state concerned.
Dissemination	Raising awareness among key stakeholders and strengthening alert and response capacity for public health emergencies.	Preparation and dissemination of a news bulletin compiling relevant national and international rumors and events to support surveillance of potential public health emergencies. Sharing of information with managers, technical units, the Cievs Network, and health professionals.

a "Signals" are data and/or information on events that represent a potential risk to human health and may include, for example, the occurrence of unusual cases or deaths, an unusual presentation of a known disease, a sudden increase in cases or deaths, or changes in the clinical and epidemiological profile of a disease. Not all "signals" indicate outbreaks or events with the potential to become public health emergencies; however, every "signal" should be assessed and verified carefully and systematically.

Mass gathering surveillance

The early detection of health risks is one of the primary objectives of mass gathering surveillance. To mitigate these risks, it is essential to implement surveillance systems that use technologies such as mobile applications and online platforms to collect and analyze data in real time^15^ ^,^ ^16^. 

A key strategy is the Integrated Health Joint Operations Center (*Centro Integrado de Operações Conjuntas da Saúde*, CIOCS), which may be activated during mass gatherings to monitor potential public health emergencies. It may be activated at the national level by the Ministry of Health (*MoH*) or at the local level by state and municipal health departments, depending on the event's complexity and scope. The Center coordinates actions across different levels of government and sectors, ensuring an integrated response, the rapid identification of emergencies, and efficient communication to support decision-making^15^.

Wastewater surveillance

Wastewater surveillance requires planning and coordination to generate information that supports public health decision-making. For its implementation, the following are recommended^17^ ^,^ ^18^: defining guidelines for monitoring priority pathogens and strengthening the laboratory network; establishing epidemiological indicators, monitoring frequency, and alert thresholds; using mathematical models for data analysis; selecting strategic sampling sites based on population density and epidemiological risk; and maintaining an integrated database with dashboards for real-time monitoring. The strategy should include transparent risk communication to ensure that authorities and the public receive timely and clear information.

Community-based event surveillance

Community-based event surveillance has emerged to address an important gap in traditional health surveillance systems: the ability to detect early signals and events that have not yet reached health services or formal reporting systems. It establishes a direct, continuous, and sensitive channel of interaction with communities, enabling the identification of changes perceived within the territory before they develop into outbreaks, disasters, or larger-scale emergencies. 

Unlike traditional mechanisms, it does not rely exclusively on official records or healthcare-seeking behavior, thereby facilitating the identification of situations that might otherwise go undetected without community reporting. Its application is particularly relevant in remote areas, among populations in situations of vulnerability, in cross-border regions, and in contexts related to the One Health approach, including zoonotic diseases, animal deaths, environmental changes, and disasters that have the potential to affect public health^19^. 

This surveillance strategy is especially relevant in settings where there are barriers to accessing health services, low healthcare-seeking behavior, or the need to monitor specific situations that cannot be adequately tracked solely through traditional notification mechanisms. This strengthens the sensitivity of surveillance systems and enhances the capacity for timely response to potential public health emergencies.

In the Brazilian context, community-based event surveillance can be strengthened through integration with community health workers, endemic disease control workers, multidisciplinary Indigenous health teams, and environmental health surveillance professionals, who have a strong territorial presence and in-depth knowledge of local dynamics. This close connection, combined with the active role of communities, enhances the capacity to recognize early changes in health patterns and environmental risks, enabling earlier alerts and improving emergency response. 

For this strategy to achieve its full potential, however, it is essential to strengthen intra- and interinstitutional integration across different areas of the health sector and other strategic sectors, such as environmental agencies, civil protection and defense, and animal health surveillance, and to promote territorial approaches aligned with the One Health perspective.

Participatory surveillance

Participatory surveillance is an approach that involves the proactive engagement of the population in the continuous generation of information on health events within their territories. Technologies such as mobile applications and online platforms facilitate data collection and submission, making the process more accessible and transparent to the public^20^. Participatory surveillance strategies can be successfully applied in a variety of settings, including mass gatherings, enabling visitors and residents to report information in real time^13^. 

It is important to emphasize that the data collected are considered unofficial and should be verified for their accuracy and relevance. Nevertheless, they enable timely initiation of investigations and support decision-making. 

Hospital-based epidemiological surveillance

hospital-based epidemiological surveillance plays a strategic role in the early detection of and response to events of public health importance within healthcare facilities. Hospital Epidemiology Centers integrate healthcare services into surveillance networks by monitoring hospital morbidity and mortality, conducting active case-finding, conducting epidemiological investigations, and reporting notifiable diseases, health conditions, and public health events through official information systems.

These Centers maintain ongoing coordination with the National hospital-based epidemiological Surveillance Network (*Rede Nacional de Vigilância Epidemiológica Hospitalar*, Renaveh), the National Strategic Health Surveillance Information Center Network (*Rede de Centros de Informações Estratégicas em Vigilância em Saúde*, CIEVS), and other surveillance structures, thereby contributing to the timely detection of public health emergencies. They also work in partnership with Hospital Infection Control Committees (*Comissões de Controle de Infecção Hospitalar*, CCIH) and Patient Safety Centers (*Núcleos de Segurança do Paciente*, NSP) to prevent and control healthcare-associated infections and adverse events.

Monitoring and risk assessment of public health emergencies

Monitoring and risk assessment are essential components of surveillance strategies, as they enable systematic data analysis to detect changes in the epidemiological profile, identify risks, and support timely decision-making. Regardless of the approach adopted, surveillance involves the continuous observation, recording, analysis, and feedback of information to guide prevention, mitigation, preparedness, and emergency response activities.

Risk assessment is a systematic process of collecting, analyzing, and documenting information to provide an up-to-date understanding of risks within a specific period, population, and location. It uses a risk matrix based on criteria for the likelihood of occurrence and potential public health impact, taking into account the clinical and epidemiological spectrum of the disease, pressure on health services, local response capacity, and the risk of spread to other locations^21^ ^,^ ^22^.

Potential public health emergencies are routinely monitored by the Public Health Event Monitoring Committee (*Comitê de Monitoramento de Eventos de Saúde Pública*, CME ), which meets weekly to monitor rumors and relevant national and international events, conduct technical discussions, and support decision-making. The participation of representatives from different surveillance and healthcare areas, as well as partner institutions and external stakeholders, contributes to more comprehensive analyses.

Prevention, preparedness, and control measures

The set of measures for the prevention, preparedness, and control of public health emergencies is broad and encompasses continuous and interdependent actions carried out before, during, and after emergencies. These actions cover different dimensions, including governance, planning and coordination, human and financial resources, information and knowledge management, risk communication, and the organization of health services^23^.

Six strategic components are highlighted below as priorities within the context of the SUS, while recognizing that they do not encompass all actions required for public health emergency management. 

Professional training: The Brazilian MoH continuously invests in workforce capacity building through programs such as the Field Epidemiology Training Program (*Programa de Treinamento em Epidemiologia Aplicada aos Serviços do Sistema Único de Saúde*, EpiSUS)^24^, aimed at developing field epidemiology competencies at three levels (frontline, intermediate, and advanced), and the Public Health Emergencies Training Program (*Programa de Formação para as Emergências em Saúde Pública*, Profesp)^25^. These and other initiatives help establish teams capable of responding to public health emergencies while supporting states and municipalities according to their specific needs.

Contingency plans and standard operating procedures: The development and regular updating of contingency plans constitute a strategic component of preparedness, guiding responses in line with risk escalation and the impacts associated with each threat^26^. These plans should define operational phases, activation criteria, and institutional responsibilities. In conjunction with these plans, standard operating procedures promote process standardization, consistency of actions, and improved implementation quality, while facilitating workforce training and continuous improvement^27^. To support the development of contingency plans, the Brazilian Ministry of Health Contingency Planning Guide^28^ serves as the guiding framework within the SUS. 

Outbreak investigation: A timely response to outbreaks and other epidemiological events is essential to prevent severe outcomes. The Brazilian MoH has developed a methodological guide presenting the ten steps of outbreak investigation^29^, which systematizes the process. This enables investigations to be conducted more efficiently and consistently, in accordance with national and international recommendations.

Situation Rooms and Emergency Operations Centers: Situation Rooms function as epidemiological intelligence hubs, bringing together information from multiple sources to support timely decision-making, issue alerts, and guide preventive actions. Emergency Operations Centers are temporarily activated during critical situations to structure response coordination across functional areas in accordance with the Incident Command System^30^. These structures promote intersectoral integration, the effective use of data, and the rapid mobilization of resources.

Intra-action and after-action reviews: The systematic use of these reviews fosters a culture of learning and continuous improvement^31^ ^,^ ^32^. By identifying good practices and gaps, these tools provide evidence to strengthen future responses. In 2025, the MoH published the Brazilian Ministry of Health After Action Review Guide^33^, which systematizes the concepts, methods, and tools to be used.

Risk communication and community engagement: Risk communication and community engagement constitute a cross-cutting component of all public health emergency management, involving the timely exchange of reliable information, ongoing dialogue with communities, and the joint development of solutions^8^ ^,^ ^34^. In addition to supporting decision-making, risk communication helps build trust, prevent stigmatization, and counter misinformation. When guided by the principles of equity and human rights, risk communication and community engagement ensure that populations in situations of vulnerability have appropriate access to information and can actively participate in emergency response activities^35^ ^,^ ^36^.

## Applicability to the SUS 

The occurrence of emergencies of different types highlights the need for the continuous strengthening of institutional and operational capacities to ensure the timely and effective management of these events^37^. In light of the challenges posed by public health emergencies, several opportunities have emerged to strengthen this surveillance. 

The integration of information systems is highlighted, with the development and implementation of integrated real-time data platforms^38^. They also include workforce training and capacity building, as well as^1^ ^,^ which strengthens the infrastructure and logistics of surveillance units.^37^. In addition, emphasis is placed on promoting intersectoral coordination through cooperation mechanisms among the sectors involved in emergency management. Finally, improving risk communication is advanced by developing strategies that incorporate the cultural, linguistic, and social characteristics of affected populations, ensuring that information is accessible, accurate, transparent, and timely.

Public health emergency surveillance must address several challenges. Among these, social inequalities and the multiple vulnerabilities affecting territories and populations are particularly important. Equity, a guiding principle of the National Health Surveillance Policy^4^, constitutes a fundamental pillar for ensuring effective, fair, and culturally responsive actions. The incorporation of equity is essential to protect lives and mitigate the disproportionate impacts experienced by populations that have historically been excluded or underserved. This requires recognizing the social and cultural diversity of territories, improving the production of data that make historically underserved populations visible, strengthening dialogue with communities and local leaders, and enhancing risk communication guided by human rights principles. 

Health inequities resulting from violence and processes of social inequality affect different population groups, each of which requires attention to its specific needs. These include Black, *Quilombola*, and Indigenous populations; rural, forest, and waterside communities; international migrants; refugees; stateless persons; LGBTQIAPN+ populations; individuals deprived of liberty; romani populations; among others.^39^. 

## Concluding Remarks 

The guidelines presented in this document provide a framework for organizing public health emergency surveillance within the SUS by integrating strategies including indicator-based and event-based surveillance, continuous monitoring, risk assessment, and preparedness and response measures. By integrating the various elements described in these guidelines, public health emergency surveillance is established as a strategic instrument for protecting lives and promoting timely, evidence-informed, territorially responsive, and socially just responses.
