# Supplementary material for: National Guidelines for Public Health Emergency Surveillance within the Brazilian Unified Health System
Source: Epidemiol Serv Saude. 2026 Oct 12;35:e20260071. doi: 10.1590/S2237-96222026v35e20260071.en (PMC13671082; doi:10.1590/S2237-96222026v35e20260071.en)
Supplement: Translation

# Translation: Diretrizes Nacionais para Vigilância das Emergências em Saúde Pública no âmbito do Sistema Único de Saúde

**DOI:** 10.1590/S2237-96222026v35e20260071.pt

**Authors:** Raquel Proença, Camila Cunha Maia Nogueira Nunes, Alessandro Pecego Martins Romano, Camila Pinto da Silva, Carlos Henrique Michiles Frank, Felipe Soares de Moraes, Francisco Silvanei dos Santos Gonçalves, Gabriel dos Santos Bandeira, Igor de Assis Rodrigues, João Roberto Cavalcante, Jose Lucas Pinho da Fonseca, Lucio Campello Silva, Magda Machado Saraiva Duarte, Maiara Almeida Maia, Mariana Sanches de Mello, Marina Pissurno do Nascimento, Otto Henrique Nienov, Paula Orofino Moura Costa, Rebeca Cristine Campos Martins, Sabrina Araújo de Melo, Vanessa de Paula Ferreira, Vinícius de Souza Casaroto, Daniel Roberto Coradi de Freitas, Taynná Vernalha Rocha Almeida, Marcio Henrique de Oliveira Garcia, Edenilo Baltazar Barreira

**Affiliations:** 1 Ministério da Saúde, Departamento de Emergências em Saúde Pública, Brasília, DF, Brasil

**Keywords:** Emergências em Saúde Pública, Saúde Pública, Vigilância em Saúde Pública, Sistema Único de Saúde, Diretrizes

## Abstract

**Objetivo:**

Descrever as diretrizes nacionais para a vigilância das emergências em saúde pública no âmbito do Sistema Único de Saúde.

**Diretrizes:**

A intensificação de eventos sanitários, ambientais e tecnológicos tem ampliado a ocorrência de emergências em saúde pública, o que exige sistemas capazes de detectar precocemente ameaças, avaliar riscos e mobilizar respostas oportunas. A vigilância das emergências integra o campo da vigilância em saúde e compreende estratégias como vigilância baseada em indicadores, vigilância baseada em eventos, vigilância epidemiológica hospitalar, vigilância de eventos de massa, vigilância de águas residuais, vigilância de eventos baseada em comunidades e vigilância participativa. Essas estratégias complementam-se e devem ser acompanhadas de monitoramento contínuo e avaliação sistemática de risco e incorporar abordagens multissetoriais, comunicacionais e de saúde única. O fortalecimento da vigilância das emergências envolve a integração de sistemas de informação, a qualificação da força de trabalho, a institucionalização de planos de contingência e procedimentos operacionais, a ativação de salas de situação e centros de operações, a investigação oportuna de surtos, a comunicação de risco e a realização de avaliações intra e pós-evento. Elementos como desigualdades territoriais, vulnerabilidades sociais e especificidades populacionais influenciam a exposição ao risco, a capacidade de detecção e os impactos das emergências, o que demanda inclusão explícita da equidade na vigilância.

**Conclusão:**

As diretrizes contribuem para consolidar a vigilância das emergências como componente estruturante da preparação e resposta do Sistema Único de Saúde, reforçando abordagens participativas, integradas e orientadas pela equidade para proteger a saúde e reduzir danos.

## Introdução 

As populações estão continuamente sob risco de doenças e agravos que podem desencadear surtos, epidemias e pandemias, além de potenciais desastres e desassistência à população. A orientação nacional da vigilância das emergências visa favorecer a tomada de decisões para fortalecer a preparação, o alerta precoce e a incorporação de novas estratégias, além de cumprir os compromissos estabelecidos no Regulamento Sanitário Internacional^1^ ^,^ ^2^.

A frequência, a escala e a complexidade das emergências têm aumentado; impulsionadas por impactos ambientais e mudanças climáticas, contribuem para o surgimento e o ressurgimento de doenças, bem como para desastres e eventos extremos^3^. Esse contexto pode ser potencializado por desassistência resultante de sistemas de saúde enfraquecidos ou sobrecarregados, o que agrava ainda mais as desigualdades sanitárias, econômicas e sociais^3^. 

A vigilância das emergências em saúde pública integra o escopo da vigilância em saúde e corresponde ao processo contínuo e sistemático de detecção, verificação, monitoramento, avaliação de risco e comunicação de risco de eventos de saúde pública que possam se constituir como potenciais emergências^4^. Essa vigilância requer abordagem multissetorial direcionada a doenças, agravos e eventos de saúde pública que apresentam maior risco para o território. Deve considerar especificidades da localidade, o que inclui perfil epidemiológico, fatores socioeconômicos, distribuição e estrutura populacional e outros elementos relevantes para a abordagem de saúde única. 

Destaca-se a importância de instituir estratégias de vigilância específicas voltadas para populações diretamente expostas e afetadas, assim também como para populações vulnerabilizadas^4^. Diante das emergências, medidas de prevenção, controle e contenção de riscos, danos e agravos à saúde pública devem ser estabelecidas em um processo contínuo no âmbito do Sistema Único de Saúde (SUS), em todos os níveis de gestão^4^.

São objetivos da vigilância das emergências em saúde pública: detectar, verificar e notificar precocemente eventos de saúde pública; avaliar riscos e monitorar os eventos considerando uma abordagem multirriscos; estabelecer fluxos de comunicação rápida diante de potenciais emergências; e articular estratégias de cooperação tripartite do SUS, promovendo a implementação da Política Nacional de Vigilância em Saúde nas ações de vigilância das emergências^4^.

As lacunas na vigilância do nível local ao global deixam o mundo vulnerável. Para enfrentar essas vulnerabilidades, a vigilância em saúde colaborativa surge como uma nova abordagem que combina técnicas tradicionais com iniciativas inovadoras visando proteger a saúde^3^. No Brasil, observa-se a incorporação progressiva do modelo colaborativo por meio da articulação de diferentes estratégias, como a vigilância sindrômica, genômica, de águas residuais e o monitoramento de rumores. A detecção precoce permite que os eventos de saúde pública sejam controlados rapidamente de maneira a mitigar os riscos e evitar potenciais emergências^2^. 

Este artigo teve como objetivo descrever as diretrizes nacionais para a vigilância das emergências em saúde pública.

## Princípios do SUS e Fundamentação Conceitual 

As diretrizes aqui apresentadas foram fundamentadas nos princípios e nas diretrizes que orientam a organização e o funcionamento do SUS. Destacaram-se a universalidade, a integralidade e a equidade, que orientam a formulação de políticas públicas e a organização das ações de saúde, incluindo aquelas voltadas à preparação, à vigilância e à resposta às emergências^5^. 

A universalidade assegura o acesso de toda a população às ações e aos serviços de saúde; a integralidade pressupõe a articulação de ações que considerem as necessidades de saúde de forma ampliada; e a equidade orienta a priorização de grupos e territórios vulnerabilizados, reconhecendo desigualdades sociais, econômicas e culturais que impactam a saúde^5^. Esses princípios devem se expressar de forma transversal nas estratégias de vigilância das emergências, orientando desde a detecção precoce até a resposta e a recuperação.

As duas principais estratégias de vigilância de emergências são a vigilância baseada em indicadores e a vigilância baseada em eventos (Figura 1)^2^ ^,^ ^4^ ^,^ ^6^ ^,^ ^7^. A vigilância baseada em indicadores é uma estratégia de monitoramento da saúde pública que se baseia na análise de indicadores quantitativos para avaliar o estado de saúde das populações, o que possibilita a detecção precoce de surtos, a identificação de tendências epidemiológicas e a avaliação da eficácia de intervenções em saúde pública^8^. 

A vigilância baseada em eventos é uma estratégia de detecção precoce, que envolve a coleta rápida e contínua, a avaliação e a interpretação de informações não estruturadas para detectar eventos de saúde pública ou as situações que podem representar um risco, que geralmente não são capturadas ou são identificadas posteriormente nos sistemas tradicionais de informação^2^ ^,^ ^6^ ^,^ ^7^. Essas informações incluem rumores provenientes de fontes formais (oficiais), como relatórios oficiais de serviços de saúde, e fontes informais (não oficiais), como notícias propagadas pela mídia, pela comunidade e por organizações não governamentais^2^ ^,^ ^6^ ^,^ ^7^. 

A vigilância para eventos de massa ocorre durante grandes aglomerações de pessoas, como festas populares, competições esportivas, shows e outros eventos de grande porte. O monitoramento desses eventos envolve uma série de ações voltadas para vigilância e controle de riscos à saúde e busca garantir uma resposta rápida e eficaz em caso de emergências^9^ ^,^ ^10^.

**Figura 1:** Componentes da vigilância das emergências em saúde pública. Brasil, 2026

A vigilância de águas residuais no contexto das emergências permite monitorar patógenos no nível populacional por meio da análise de águas residuais. As águas residuais são aquelas águas doces descartadas após a sua utilização em diversas atividades humanas realizadas em residências, comércios, indústrias e serviços municipais, incluindo águas pluviais^11^. 

A vigilância de eventos baseada em comunidades consiste em uma abordagem participativa de vigilância em saúde voltada à identificação e à comunicação precoce de sinais, rumores ou eventos de saúde pública, a partir da atuação de membros da própria comunidade^12^. Fundamentada na valorização do conhecimento territorial e da percepção comunitária sobre alterações incomuns no cotidiano, essa estratégia possibilita captar informações que muitas vezes não são detectadas pelos sistemas formais de vigilância.

A vigilância participativa refere-se ao monitoramento de saúde em que a população desempenha um papel ativo no fornecimento de informações em seus territórios^13^. A população é a primeira a observar e sentir os efeitos das doenças e dos agravos, e isso permite alertar rapidamente os primeiros sinais às autoridades de saúde, o que possibilita uma resposta mais ágil e proporcional.

A vigilância epidemiológica hospitalar nasceu da necessidade de descentralizar as atividades nos estabelecimentos de saúde, através dos Núcleos Hospitalares de Epidemiologia. As doenças, os agravos e os eventos de saúde pública identificados nos serviços de saúde devem ser prontamente comunicados pelos núcleos às demais redes, aos setores e aos serviços que desenvolvem atividades de vigilância epidemiológica em âmbito local e nacional.

Os principais conceitos no âmbito das emergências foram apresentados na Tabela 1.

**Tabela 1:** Conceitos-chave das emergências em saúde pública no contexto das diretrizes nacionais de vigilância das emergências no âmbito do Sistema Único de Saúde

Conceito	Definição
Evento de saúde pública^40^	Situação que pode constituir potencial ameaça à saúde pública, como a ocorrência de surto ou epidemia, doença ou agravo de causa desconhecida, alteração no padrão clínico-epidemiológico das doenças conhecidas. Consideram-se o potencial de disseminação, a magnitude, a gravidade, a severidade, a transcendência e a vulnerabilidade, bem como as epizootias ou os agravos decorrentes de desastres ou acidentes.
Emergência em saúde pública^41^	Situação que demanda o emprego urgente de medidas de prevenção, de controle e de contenção de riscos, de danos e de agravos à saúde pública.
Emergência em saúde pública de importância nacional (Espin)^41^	Emergência em saúde pública que, nos termos do Decreto nº 7.616, de 17 de novembro de 2011, poderá ser declarada em virtude da ocorrência das seguintes situações: epidemiológicas, de desastres ou de desassistência à população.
Emergência em saúde pública de importância internacional (Espii)^1^	Evento extraordinário que, nos termos do Regulamento Sanitário Internacional, é determinado como: risco para a saúde pública para outros Estados, devido à propagação internacional de doença; e, potencialmente, exige resposta internacional coordenada.
Surto^29^	Situação em que há aumento acima do esperado de casos da doença, do agravo ou do evento de saúde pública em área ou grupo de pessoas específico, em um determinado período.
Epidemia^29^	Situação em que a doença envolve grande número de pessoas e atinge larga área geográfica.
Pandemia^42^	Epidemia que ocorre em todo o mundo ou em área muito vasta, que cruza fronteiras internacionais e que, geralmente, afeta muitas pessoas.
Desastres naturais^43^	Eventos causados por processos ou fenômenos naturais (hidrológicos, climatológicos, meteorológicos e geológicos), que podem implicar perdas humanas ou outros impactos à saúde, danos ao meio ambiente e à propriedade, que provocam interrupção dos serviços e distúrbios sociais e econômicos.
Desastres tecnológicos^43^	Eventos originados de condições tecnológicas ou industriais relacionados aos agentes químicos, biológicos, radiológicos e nucleares. Incluem acidentes, incidentes ou atividades humanas específicas que podem implicar perdas humanas ou outros impactos à saúde, além de danos ao meio ambiente e à propriedade, interrupção dos serviços e distúrbios sociais e econômicos. Podem ocorrer de forma intencional ou não.
Desassistência à população^41^	Evento que, devidamente reconhecido mediante a decretação de emergência ou calamidade pública pelo ente federado afetado, que coloque em risco a saúde dos cidadãos por incapacidade ou insuficiência de atendimento à demanda e que extrapole a capacidade de resposta das direções estadual e municipal do Sistema Único de Saúde.

## Diretrizes 

As diretrizes a seguir estão orientadas pelos princípios e pelas diretrizes do SUS, que se expressam, de forma transversal, nas diferentes estratégias de vigilância das emergências em saúde pública.

Vigilância baseada em indicadores 

A vigilância baseada em indicadores fundamenta-se no uso de indicadores como medidas-síntese do estado de saúde da população e do desempenho dos serviços de saúde e pode variar desde a contagem direta de casos até o cálculo de proporções, razões e taxas^14^. Ela pode ser aplicada: na detecção de surtos, para monitorar indicadores que auxiliam na identificação e na resposta rápida; na avaliação de programas de saúde, para mensurar as intervenções; no planejamento de serviços de saúde, para informar a alocação de recursos e a distribuição de serviços de saúde, com base nas necessidades identificadas; e na vigilância de epidemiológica, para monitorar a prevalência, a incidência e os determinantes associados a doenças. 

**Tabela 2:** Etapas do ciclo da vigilância baseada em indicadores no contexto das diretrizes nacionais para vigilância das emergências em saúde pública no âmbito do Sistema Único de Saúde

Etapa	Componentes
Coleta de dados	Identificação e captura de dados (sistemas de registros e outras fontes). Garantia de qualidade, completude e tempestividade.
Análise de dados	Processamento dos dados. Identificação de padrões, tendências e anomalias. Uso de ferramentas estatísticas e softwares de análise adequados.
Interpretação	Contextualização dos achados no cenário epidemiológico. Consideração de fatores locais e políticas de saúde. Análise crítica dos resultados.
Disseminação	Comunicação dos resultados à rede de vigilância e gestores. Compartilhamento para profissionais de saúde e a população, quando pertinente. Comunicação técnica clara e oportuna.

Essa vigilância segue um ciclo contínuo de coleta, análise, interpretação e disseminação de dados (Tabela 2). 

A coleta de dados pode ser realizada por meio de diversas fontes, como registros hospitalares, sistemas de informação em saúde, inquéritos populacionais e notificações de doenças. Adicionalmente, devem ser considerados indicadores relacionados à capacidade dos serviços de saúde, como taxas de ocupação de leitos e demanda por atendimentos, que podem sinalizar situações de risco de desassistência.

Vigilância baseada em eventos 

A vigilância baseada em eventos estrutura-se em um fluxo que organiza o tratamento dos sinais capturados até que se convertam em informações verificadas e úteis. Esse fluxo compreende cinco etapas: detecção, triagem, avaliação, verificação e disseminação (Tabela 3).

Essa vigilância pode se expressar por meio de distintas modalidades operacionais, que preservam as etapas estruturantes anteriormente descritas, ao mesmo tempo em que ampliam sua capilaridade e fortalecem a identificação oportuna de eventos.

Desastres naturais e tecnológicos

No contexto dos desastres naturais e tecnológicos, a vigilância baseada em eventos deve contemplar o monitoramento de dados, informações e alertas de risco, o que inclui previsões meteorológicas. Devem ser considerados os registros provenientes de órgãos de proteção e defesa civil e de instituições ambientais, além de acompanhar as áreas suscetíveis à ocorrência de desastres, as populações expostas e a rede de saúde nesses territórios. 

O monitoramento do estoque estratégico de medicamentos, insumos e equipamentos que podem ser mobilizados durante emergências integra esse processo, assim como o estabelecimento da captura de rumores e o envio de alertas aos órgãos competentes. A caracterização do território permite identificar riscos associados a enchentes, inundações, movimentos de massa e falhas em barragens ou infraestruturas críticas. A avaliação de impacto dos desastres na saúde, a análise de situação sobre doenças transmissíveis e não transmissíveis e a caracterização das populações afetadas e vulnerabilizadas são etapas centrais.

Nos desastres por emergências químicas, biológicas, radiológicas e nucleares, a vigilância baseada em eventos inclui a detecção de eventos que possam desencadear emergências em saúde pública, a caracterização do território e a identificação de fatores de risco relacionados a esses agentes. Também envolve o mapeamento de hospitais e redes de referência e a articulação intersetorial para garantir respostas oportunas e integradas. Inclui captura de rumores, avaliação de impacto, análise de dados, caracterização das populações afetadas e avaliação da situação de saúde.

**Tabela 3:** Etapas do ciclo da vigilância baseada em eventos no contexto das diretrizes nacionais para vigilância das emergências em saúde pública no âmbito do Sistema Único de Saúde

Etapas	Descrição	Ferramenta/processo
Detecção	Identificação de "sinais"^a^ em sites e ferramentas de pesquisa na internet, reportagens na mídia e nos jornais.	Epidemic Intelligence from Open Sources
Triagem	Triagem das informações obtidas para a avaliação inicial.	Leitura do título e do conteúdo da notícia, identificando: se há informações que indicam alterações do perfil clínico-epidemiológico de doença conhecida; se há relatos de irregularidades de produtos e serviços; o possível surgimento de doença inusitada ou desconhecida; eventos de saúde animal que podem ter impacto na saúde humana; e desastres naturais ou tecnológicos com potencial impacto na saúde pública, ou de desassistência.
Avaliação	Avaliação das informações coletadas e classificadas quanto à sua relevância. Determinação se um rumor tem potencial para se tornar uma emergência em saúde pública.	Classificação como relevante ou não relevante, que utiliza como base: extensão geográfica; características do evento do rumor; disponibilidade assistencial; e transcendência e capacidade de resposta.
Verificação	Para os rumores classificados como relevantes: verificação da veracidade; e coleta de demais informações relevantes em fontes oficiais. Quando um sinal é verificado, ele se torna um "evento". Os eventos devem desencadear ou incitar algum nível de resposta, o que inclui realizar investigação e adotar medidas de controle.	Rumores locais: verifica-se com atores locais, como áreas técnicas e instituições. Rumores nacionais: verifica-se com as áreas técnicas responsáveis pela doença, pelo agravo ou pelo evento de saúde pública e com as unidades da Rede Nacional dos Centros de Informações Estratégicas em Vigilância em Saúde (Cievs). Rumores internacionais: o Cievs Nacional, enquanto centro operacional para o Ponto Focal Nacional para o Regulamento Sanitário Internacional, verifica as informações ou solicita informações adicionais ao estado-membro da Organização Mundial da Saúde.
Disseminação	Sensibilizar os atores-chave e aprimorar a capacidade de alerta e resposta às emergências em saúde pública.	Confecção e divulgação do *clipping* de notícias, que reúne os rumores e os eventos nacionais e internacionais relevantes para apoiar a vigilância de potenciais emergências em saúde pública. Compartilhamento com os gestores, as áreas técnicas, a Rede Cievs e os profissionais da saúde.

a Os "sinais" são dados e/ou informações sobre eventos que representam um risco potencial para a saúde humana, podendo consistir, por exemplo, em ocorrência de casos ou óbitos incomuns, apresentação de padrão incomum de doença conhecida, aumento repentino de casos ou óbitos ou mudanças no perfil clínico-epidemiológico de uma doença. Nem todos os "sinais" são indicações de surtos ou eventos com potencial para emergência em saúde pública, mas cada "sinal" deve ser avaliado e verificado cuidadosa e sistematicamente.

Vigilância para eventos de massa

A detecção precoce de riscos à saúde é um dos principais objetivos do monitoramento de eventos de massa. Para mitigar esses riscos, é fundamental implementar sistemas de vigilância que utilizem tecnologias, como aplicativos móveis e plataformas on-line, para coletar e analisar dados em tempo real^15^ ^,^ ^16^. 

Uma estratégia central é o Centro Integrado de Operações Conjuntas da Saúde, que pode ser ativado em eventos de massa para monitorar potenciais emergências. Sua ativação ocorre em nível nacional pelo Ministério da Saúde (MS) ou em nível local pelas secretarias estaduais e municipais, conforme a complexidade e a abrangência do evento. Ele coordena ações entre diferentes níveis de governo e setores e garante resposta integrada, rápida identificação de emergências e comunicação eficiente para apoiar a tomada de decisão^15^.

Vigilância de águas residuais

A vigilância de águas residuais exige planejamento e coordenação para gerar informações que apoiem a tomada de decisão em saúde pública. Para sua implementação, recomenda-se^17^ ^,^ ^18^: definir diretrizes de monitoramento de patógenos de interesse e fortalecer a rede laboratorial; estabelecer indicadores epidemiológicos, frequência de monitoramento e limiares de alerta; utilizar modelos matemáticos para as análises; selecionar pontos de coleta estratégicos considerando densidade populacional e risco epidemiológico; e manter banco de dados integrado com painéis para acompanhamento em tempo real. A estratégia deve incluir comunicação de risco transparente, o que garante que autoridades e população sejam informadas de forma oportuna e clara.

Vigilância de eventos baseada em comunidades

A vigilância de eventos baseada em comunidades surge para preencher uma lacuna importante dos sistemas tradicionais de vigilância em saúde: a capacidade de identificar precocemente sinais e eventos que ainda não chegaram aos serviços de saúde ou aos sistemas formais de notificação. Ela estabelece um canal direto, contínuo e sensível de interação com as comunidades, o que permite captar mudanças percebidas no território antes que se consolidem como surtos, desastres ou emergências de maior magnitude. 

Diferentemente dos mecanismos tradicionais, não depende exclusivamente de registros oficiais ou da procura por atendimento, e isso favorece a identificação de situações que poderiam permanecer invisíveis caso a escuta comunitária fosse desconsiderada. Sua aplicação é especialmente relevante em áreas remotas, populações vulnerabilizadas, regiões transfronteiriças e cenários relacionados à abordagem de Uma Só Saúde, o que inclui zoonoses, mortes de animais, alterações ambientais e desastres com potencial impacto à saúde coletiva^19^. 

Essa vigilância mostra-se especialmente relevante em contextos nos quais há barreiras de acesso aos serviços de saúde, baixa procura por atendimento ou necessidade de monitoramento de situações específicas que não podem ser acompanhadas exclusivamente pelos mecanismos tradicionais de notificação. Isso fortalece a sensibilidade dos sistemas de detecção e amplia a capacidade de resposta oportuna diante de potenciais emergências.

No contexto brasileiro, a vigilância de eventos baseada em comunidades pode ser fortalecida pela integração com agentes comunitários de saúde, agentes de combate às endemias, equipes multiprofissionais da saúde indígena e profissionais da vigilância em saúde ambiental, que possuem forte inserção territorial e conhecimento aprofundado das dinâmicas locais. Essa proximidade, associada ao protagonismo das comunidades, amplia a capacidade de reconhecer precocemente alterações nos padrões de saúde e riscos ambientais, antecipando alertas e qualificando a resposta às emergências. 

Para que essa estratégia alcance seu potencial, entretanto, é fundamental fortalecer a integração intra e interinstitucional entre diferentes áreas do setor saúde e outros setores estratégicos, como meio ambiente, defesa civil e vigilância animal, promovendo abordagens territorializadas e alinhadas à perspectiva de Uma Só Saúde.

Vigilância participativa

A vigilância participativa consiste em uma abordagem que envolve o engajamento proativo da população na geração contínua de informações sobre eventos de saúde em seus territórios. Tecnologias como aplicativos móveis e plataformas on-line facilitam a coleta e o envio de dados, o que torna o processo mais acessível e transparente à população^20^. Estratégias de vigilância participativa podem ser utilizadas com sucesso em diferentes contextos, incluindo eventos de massa, permitindo que visitantes e residentes reportem informações em tempo real^13^. 

É importante ressaltar que os dados coletados são considerados não oficiais e devem ser verificados quanto à sua veracidade e relevância. Ainda assim, permitem iniciar investigações de forma oportuna e apoiar a tomada de decisão. 

Vigilância epidemiológica hospitalar

A vigilância epidemiológica hospitalar desempenha papel estratégico na detecção precoce e na resposta a eventos de interesse em saúde pública no ambiente hospitalar. Os Núcleos Hospitalares de Epidemiologia atuam na integração dos serviços de saúde às redes de vigilância, realizando monitoramento da morbimortalidade hospitalar, busca ativa, investigação epidemiológica e notificação de doenças, agravos e eventos de notificação compulsória nos sistemas oficiais.

Os núcleos mantêm articulação permanente com a Rede Nacional de Vigilância Epidemiológica Hospitalar, a Rede de Centros de Informações Estratégicas em Vigilância em Saúde e as demais estruturas de vigilância, o que contribui para a detecção oportuna de emergências, além de atuar em parceria com as Comissões de Controle de Infecção Hospitalar e os Núcleos de Segurança do Paciente na prevenção e no controle de infecções e eventos adversos.

Monitoramento e avaliação de risco de emergências em saúde pública

O monitoramento e a avaliação são componentes essenciais das estratégias de vigilância, pois permitem a análise sistemática de dados, para detectar alterações no perfil epidemiológico, identificar riscos e tomar a decisão oportuna. Independentemente da abordagem adotada, a vigilância pressupõe observação, registro, análise e retroalimentação de informações, orientando ações de prevenção, mitigação, preparação e resposta às emergências.

A avaliação de risco é um processo sistemático de coleta, análise e documentação de informações, o que permite a compreensão atualizada dos riscos em determinado período, população e local. Utiliza matriz de risco com critérios de probabilidade de ocorrência e impacto em saúde pública, considerando espectro clínico-epidemiológico da doença, pressão sobre serviços de saúde, capacidade de resposta local e risco de disseminação para outras localidades^21^ ^,^ ^22^.

As potenciais emergências são acompanhadas rotineiramente pelo Comitê de Monitoramento de Eventos de Saúde Pública, instância que se reúne semanalmente para monitorar rumores e eventos internacionais e nacionais relevantes, realizar discussões técnicas e apoiar decisões. Destaca-se a presença de diferentes áreas da vigilância e assistência, além de instituições parceiras e atores externos, o que garante análises mais abrangentes.

Medidas de prevenção, preparação e controle

O conjunto de medidas de prevenção, preparação e controle das emergências em saúde pública é amplo e envolve ações contínuas e interdependentes, que abrangem períodos anteriores, durante e posteriores às emergências. Essas ações compreendem diferentes dimensões, como governança, planejamento e coordenação, recursos humanos e financeiros, gestão da informação e do conhecimento, comunicação de risco e organização dos serviços de saúde^23^.

Destacam-se, a seguir, seis componentes estratégicos prioritários para o contexto do SUS, reconhecendo-se que não esgotam o conjunto de ações necessárias à gestão das emergências em saúde pública. 

Formação profissional: o MS investe continuamente na qualificação da força de trabalho por meio de programas como o Programa de Treinamento em Epidemiologia aplicada aos serviços do SUS^24^, voltado ao desenvolvimento de competências em epidemiologia de campo em três níveis (fundamental, intermediário e avançado), e o Programa de Formação para as Emergências em Saúde Pública^25^. Essas e outras iniciativas contribuem para consolidar equipes capacitadas a atuar nas emergências, além de apoiar estados e municípios segundo suas especificidades.

Planos de contingência e procedimentos operacionais padrão: a elaboração e a atualização de planos de contingência constituem eixo estratégico da preparação, orientando a resposta conforme o escalonamento do risco e dos impactos associados a cada ameaça^26^. Esses planos devem prever estágios operacionais, critérios de ativação e responsabilidades institucionais. Associados a eles, os procedimentos operacionais padrão promovem padronização de processos, consistência nas ações e maior qualidade na execução, ao mesmo tempo em que facilitam o treinamento das equipes e a melhoria contínua^27^. Para orientar a elaboração dos planos de contingência, o Guia de Elaboração de Planos de Contingência do MS^28^ atua como dispositivo norteador no âmbito do SUS. 

Investigação de surtos: a resposta oportuna a surtos e a outros eventos epidemiológicos é fundamental para prevenir desfechos graves. O MS dispõe de um guia metodológico que apresenta dez passos da investigação de surtos^29^ e sistematiza o processo investigativo. Isso permite a condução mais eficiente e padronizada, alinhada às recomendações nacionais e internacionais.

Salas de Situação e Centros de Operações de Emergência: as Salas de Situação funcionam como núcleos de inteligência epidemiológica e reúnem informações de diferentes fontes para subsidiar decisões oportunas, emitir alertas e apoiar ações preventivas. Os Centros de Operações de Emergência são ativados de forma temporária em situações críticas, estruturando a coordenação da resposta em áreas funcionais de acordo com o sistema de comando de incidentes^30^. Essas estruturas promovem integração intersetorial, uso qualificado de dados e mobilização rápida de recursos.

Avaliação intra e pós-evento: o uso sistemático da avaliação intra e pós-evento consolida a cultura de aprendizado e de aprimoramento contínuo^31^ ^,^ ^32^. Ao identificar boas práticas e lacunas, esses instrumentos oferecem subsídios para fortalecer futuras respostas. O MS publicou em 2025 o Guia de Avaliação Pós-Evento para Emergências em Saúde Pública^33^, que sistematiza conceitos, métodos e ferramentas a serem utilizados.

Comunicação de risco e engajamento comunitário: constitui eixo transversal de toda a gestão de emergências, pois envolve troca oportuna de informações confiáveis, diálogo permanente com comunidades e construção conjunta de soluções^8^ ^,^ ^34^. Além de apoiar a tomada de decisão, a comunicação de risco contribui para gerar confiança, prevenir estigmatização e combater desinformação. Quando orientados por princípios de equidade e direitos humanos, a comunicação de risco e o engajamento comunitário garantem que grupos vulneráveis tenham acesso às informações de forma adequada, participando ativamente das ações de resposta^35^ ^,^ ^36^.

## Aplicabilidade ao SUS 

A ocorrência de emergências de diferentes naturezas evidencia a necessidade de aprimoramento contínuo das capacidades institucionais e operacionais para enfrentamento oportuno e eficaz desses eventos^37^. Diante dos desafios impostos nas emergências, algumas perspectivas se delineiam para fortalecer essa vigilância. 

Destaca-se a integração dos sistemas de informação, com o desenvolvimento e a implementação de plataformas integradas de dados em tempo real^38^. Incluem-se também a capacitação e a valorização dos recursos humanos^1^ e o fortalecimento da infraestrutura e da logística das unidades de vigilância^37^. Além disso, ressalta-se a promoção da articulação intersetorial através de mecanismos de cooperação entre os setores envolvidos na gestão de emergências. Por fim, destaca-se o aprimoramento da comunicação de risco, desenvolvendo estratégias que integrem as especificidades culturais, linguísticas e sociais das populações afetadas, garantindo informações acessíveis, precisas, transparentes e oportunas.

A vigilância das emergências deve considerar diversos desafios. Entre eles, destacam-se as desigualdades sociais e as múltiplas vulnerabilidades que atravessam territórios e populações. A equidade, princípio estruturante da Política Nacional de Vigilância em Saúde^4^, constitui eixo fundamental para garantir respostas efetivas, justas e culturalmente sensíveis. Incorporar a equidade é imprescindível para proteger vidas e mitigar impactos desproporcionais em populações historicamente excluídas ou negligenciadas. Isso implica reconhecer a diversidade social e cultural dos territórios, qualificar a produção de dados que visibilizem populações historicamente negligenciadas, fortalecer o diálogo com comunidades e lideranças locais e aprimorar a comunicação de risco orientada pelos direitos humanos. 

As iniquidades em saúde produzidas pelas violências e pelos processos de desigualdade social afetam diferentes populações, que, por sua vez, demandam atenção para suas especificidades. Entre elas, estão as populações negra, quilombola e indígena, as comunidades do campo, da floresta e das águas, os migrantes internacionais, os refugiados, os apátridas, a população LGBTQIAPN+, as pessoas privadas de liberdade, os povos ciganos/romani, entre outros^39^. 

## Considerações Finais 

As diretrizes apresentadas estruturam um referencial para organizar a vigilância das emergências em saúde pública no âmbito do SUS, ao integrar estratégias como a vigilância baseada em indicadores e em eventos, o monitoramento contínuo, a avaliação de risco e as medidas de preparação e resposta. Ao integrar os diversos elementos citados nesta diretriz, a vigilância das emergências se consolida como instrumento estratégico para proteger a vida e para promover respostas oportunas, com base em evidências, territorialmente sensíveis e orientadas pela justiça social.
